# Dominant role of microglial and macrophage innate immune responses in human ischemic infarcts

**DOI:** 10.1111/bpa.12583

**Published:** 2017-12-28

**Authors:** Tobias Zrzavy, Joana Machado‐Santos, Sheren Christine, Christoph Baumgartner, Howard L. Weiner, Oleg Butovsky, Hans Lassmann

**Affiliations:** ^1^ Center for Brain Research Medical University of Vienna Austria; ^2^ Department of Neurology General Hospital Hietzing with Neurological Center Rosenhügel, Sigmund Freud University Vienna Austria; ^3^ Ann Romney Center for Neurological Diseases, Brigham and Women's Hospital, Harvard Medical School Boston MA; ^4^ Evergrande Center for Immunologic Diseases, Brigham and Women's Hospital, Harvard Medical School Boston MA

**Keywords:** granulocytes, lymphocytes, macrophages, microglia, P2RY12, Stroke, TMEM119

## Abstract

Inflammatory mechanisms, involving granulocytes, T‐cells, B‐cells, macrophages and activated microglia, have been suggested to play a pathogenic role in experimental models of stroke and may be targets for therapeutic intervention. However, knowledge on the inflammatory response in human stroke lesions is limited. Here, we performed a quantitative study on the inflammatory reaction in human ischemic infarct lesions. We found increased numbers of T‐lymphocytes, mainly CD8^+^ cells, but not of B‐lymphocytes. Their number was very low in comparison to that seen in inflammatory diseases of the central nervous system and they did not show signs of activation. Polymorphonuclear leukocytes were present in meninges and less prominently in the perivascular space in early lesions, but their infiltration into the lesioned tissue was sparse with the exception of a single case. Microglia were lost in the necrotic core of fresh lesions, their number was increased in the surrounding penumbra, apparently due to proliferation. Using TMEM119 as a marker for the resident microglia pool, macrophages in lesions were in part derived from the original microglia pool, depending on the lesion stage. Most microglia and macrophages revealed a pro‐inflammatory activation pattern, expressing molecules involved in phagocytosis, oxidative injury, antigen presentation and iron metabolism and had partially lost the expression of P2RY12, an antigen expressed on homeostatic (“resting”) microglia in rodents. At later lesion stages, the majority of macrophages showed intermediate activation patterns, expressing pro‐inflammatory and anti‐inflammatory markers. Microglia in the normal white matter of controls and stroke patients were already partly activated toward a pro‐inflammatory phenotype. Our data suggest that the direct contribution of lymphocytes and granulocytes to active tissue injury in human ischemic infarct lesions is limited and that stroke therapy that targets pro‐inflammatory microglia and macrophage activation may be effective.

## Introduction

Recent experimental studies suggest a major role of inflammatory mechanisms in ischemic or hemorrhagic stroke 33. After an acute stage of focal tissue necrosis, microglia and granulocytes are recruited into the lesions within hours to few days, followed by invasion of lymphocytes and monocytes 10, 20, 29. The activated microglia population and the recruited monocytes transform into macrophages in the lesions 46. All these changes occur in experimental models within 1–7 days after the initiating insult. Based on these observations, several therapeutic strategies have been tested aiming to reduce granulocyte and lymphocyte infiltration or blocking microglia/macrophage recruitment and/or activation, and such treatments have provided beneficial effects in experimental animal models 29. However, translation of these promising results into clinical application has not been successful so far 58.

To date, information on the nature and dynamic evolution of the inflammatory response in human ischemic infarct lesions is limited. There seem to be differences to experimental models in the global time course and extent of inflammation 38 or the contribution of granulocytes 14. Little is known regarding the contribution of microglia versus recruited monocytes and the quantity, composition and activation state of lymphocytes, microglia and macrophages within different lesions stages. We investigated the dynamics of inflammation and microglial activation to provide a basis for the design of anti‐inflammatory treatment strategies in human stroke.

## Material and Methods

### Sample characterization

**Figure 1:**
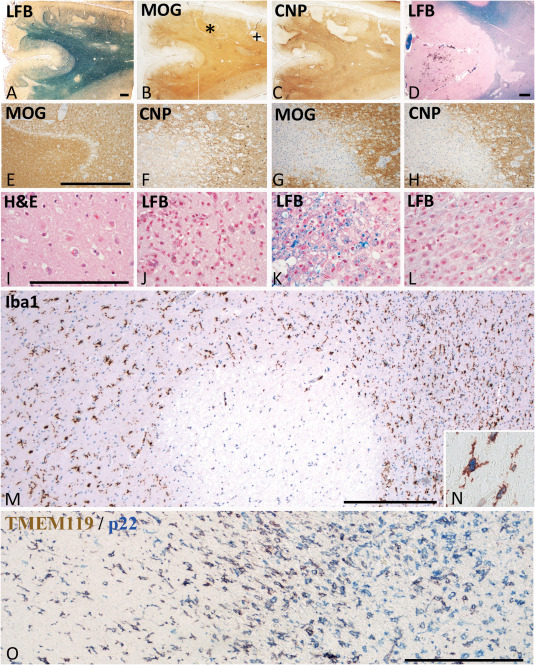
Neuropathological basis of the staging of acute ischemic infarct lesions. **A–C.** 85 year old femal patient with a stroke history of 31 days. Parietal cortex and subcortical white matter with multiple acute and subscute ischemic infarct lesions due to thrombosis of meningeal artery. Multiple focal lesions in the cortex and the subcortical white matter. Some of the lesions are in the initial stage of tissue necrosis, defined by reduced myelin staining in luxol fast blue and loss of CNPase expression, while MOG is still preserved (*); More advanced lesions show complete loss of myelin and a parallel loss of CNP and MOG (+). **D.** 85 year old female with a stroke history of 8 days; acute cortico‐subcortical ischemic infacr lesion with some hemorrhagic component in the cortical lesion part. **E,F.** Higher magnification of the lesion indicated by * in Figure 1A; the acute ischemic lesion is demarcated from the surrounding normal tissue by a band of tissue edema; within the lesion there is a complete loss of CNPase, but a preservation of MOG immunoreactivity; **G**,**H.** higher magnification of the lesion indicated by + in Figure 1; there is a complete loss of both myelin proteins (MOG in g) and CNP in h); the lesion appears hypercellular due to the macrophage infiltration present in the resorption stage; A–H. Magnification bar: 1 mm. **I.** Acute ischemic infarct lesion in the cortex of the patient, shown in Figure 1D; profound reduction of cell density in the cortex and in particular a profound loss of neurons and pale neurons with nuclear fragmentation and some apoptotic nuclei of glia cells; hematoxlyin & eosin staining; **J–L.** Different lesions in the resorption stage in a 97 year old female patient; (J) Cortical lesion characterized by a high density of activated microglia and macrophages, some of them containing early and late lipid degradation products; (K) White matter lesion in the early resorption stages with macrophages, containing luxol fast blue reactive degradation products; (L) White matter lesion in the late resorption stages with macrophages containing empty lipid vacuoles; **M,N.** Microglia reaction within and around the acute ischemic inflarct lesion, shown by * in Figure 1A; the lesion is located at the border between white and grey matter; within the lesions Iba‐1 postive cells are completely lost; at the lesion edge microglia starts to increase in density and many of these cells are double labeled with the proliferation marker PCNA (N). **O.** Double staining for TMEM119 and NADPHoxidase (p22phox) in a lesion at the early resorption stage. Microglia is found in the perilesional area (left side). At the edge of the lesion, a profound increase of microglia and macrophage like cells is present, the majority of those still being double stained for both markers. In the center of the lesions, numerous macrophages are present, which are p22phox^+^/TMEM119^–^ cells. (C–O) Magnification bar:200 μm.

Our study was performed on archival paraffin embedded autopsy tissue collected in the Center for Brain Research (Vienna). The study was approved by the Ethik Kommission of the Medical University of Vienna (EK. 535/2004/2017).It included tissue blocks, containing the brain lesions of ischemic infarcts of 16 patients and from 18 controls without neurological disease or neuropathologically detectable brain damage. Patient demographics, clinical data of disease history, confounding diseases and cause of death are presented in Table 1. Since brain inflammation in some aspects may differ between the white and grey matter, tissue blocks were selected from a broader sample of neuropathologcal specimens, which contained either lesions affecting both or included separate lesions in both compartments (Figure 1 and Table 2). Five out of eighteen controls and 8/16 patients died in the course of a concomittant systemic infection. Inflammation and microglia activation in these patients was compared with that seen in patients without evidence for systemic infectious disease.

**Table 1:** Clinical data of included patients.

Case	Details	Sex	Age	Lesion	Cause of Death	Time to death	Infection
Control 1	Control	female	30		hemoptysis		
Control 2	Control	female	36		aspiration		
Control 3	Control	male	37		cardiovascular failure		septic/pneumonia
Control 4	Control	female	39		cervix Ca		
Control 5	Control	female	42		lung Ca		
Control 6	Control	female	45		pulmonary embolism		
Control 7	Control	male	46		pulmonary embolism		
Control 8	Control	female	47		heart failure		
Control 9	Control	male	65		heart failure		
Control 10	Control	male	70		cardiovascular failure		septic/pneumonia
Control 11	Control	female	71		heart failure		
Control 12	Control	female	71		renal failure		septic
Control 13	Control	male	72		cardiovascular failure		
Control 14	Control	female	80		n.a.		
Control 15	Control	male	83		cardiovascular failure		
Control 16	Control	female	84		pulmonary embolism		
Control 17	Control	female	88		cardiovascular failure		septic/pneumonia
Control 18	Control	female	97		cardiovascular failure		septic/pneumonia
Stroke 1	Stroke	male	67	acute	aortic rupture	1	
Stroke 2	Stroke	female	88	acute	cardiac arrest	3	
Stroke 3	Stroke	male	66	acute, res	respiratory failure	8	pneumonia
Stroke 4	Stroke	male	69	acute, res	pneumonia	43	pneumonia
Stroke 5	Stroke	male	84	acute, res	pulmonary edema	1	aortic valve
Stroke 6	Stroke	female	85	acute, res	cardiopulmonary failure	8	
Stroke 7	Stroke	female	85	acute, res	pulmonary embolism	31	
Stroke 8	Stroke	female	92	acute, res, scar	respiratory failure	31	urinary tract infection
Stroke 9	Stroke	female	78	acute, scar	heart failure	14	sepsis
Stroke 10	Stroke	male	70	res	n.a.	n.a.	
Stroke 11	Stroke	male	73	res	pneumonia	73	pneumonia
Stroke 12	Stroke	female	86	res	cardiac arrest	n.a.	urinary tract infection
Stroke 13	Stroke	female	91	res	n.a.	n.a.	n.a.
Stroke 14	Stroke	female	92	res	respiratory failure	n.a.	
Stroke 15	Stroke	male	74	res, scar	cardiopulmonary failure	240	
Stroke 16	Stroke	female	97	scar	heart failure	n.a.	pneumonia

Abbreviations: acute = acute lesion stage; res = resorption stage; scar = late cystic or scar lesion stage; n.a. = data not available; time to death = time span (days) between onset of clinical or radiological manifestations of ischemia to death; infection = infection at time of death.

**Table 2:** Neuropathological data of included stroke patients.

Case	Details	Tissue blocks	Erythrocytes	Vessel (lesion)	M. granulocytes	TOAST
Stroke 1	Stroke	10/2	+		+/−	other determined cause
Stroke 2	Stroke	4/3	+	small vessel Thomb.	+	large‐artery atherosclerosis
Stroke 3	Stroke	3/1	−	small vessel occlusion	−	small‐vessel occlusion
Stroke 4	Stroke	1/1	−		−	other determined cause
Stroke 5	Stroke	7/3	−	men. A thromb.	+	undetermined cause
Stroke 6	Stroke	3/3	+	large artery occlusion	+/−	large‐artery atherosclerosis
Stroke 7	Stroke	1/1	−	men. vessel occlusion	++	cardio embolism
Stroke 8	Stroke	1/1	+	men. vessel occlusion	+/−	large‐artery atherosclerosis
Stroke 9	Stroke	1/1	−	men. vessel occlusion	+/−	other determined cause
Stroke 10	Stroke	8/1	−	men. vessel occlusion	+/−	undetermined cause
Stroke 11	Stroke	1/1	+/−	old thrombus	−	small‐vessel occlusion
Stroke 12	Stroke	4/4	−		−	cardio embolism
Stroke 13	Stroke	2/1	−	men. vessel occlusion	−	undetermined cause
Stroke 14	Stroke	1/1	−	thrombotic recanalized vessel	−	cardio embolism
Stroke 15	Stroke	1/1	−		−	small‐vessel occlusion
Stroke 16	Stroke	3/2	−		−	undetermined cause

Summary of pathological data fo the stroke patients, including number of blocks investigated, presence or absence of perivascular erythrocytes, thrombotic occlusion of meningeal or parenchymal vessels, presence or absence of meningeal granulocyte infiltrations and clinical definition of disease according to the TOAST criteria. M. Granulocytes = granulocytes in meninges; men = meningeal.

A clear staging in relation to the time window between the onset of disease and the time of investigation of the lesions was only possible in a subset of cases, since a broad spectrum of different lesion stages can often be seen side‐by‐side within the same tissue blocks indicating recurrent new episodes of ischemia and the appearance of new lesions during the clinical observation period 38. For this reason, we determined the nature and dynamics of inflammation in lesions, staged by neuropathological criteria. For our study the following areas of interest were analysed:

- **Normal appearing white and grey matter of controls (NAWMC/NAGMC)**: This included white and grey matter areas from patients without neurological disease or focal or diffuse neuropathological changes present in sections stained with hematoxylin & eosin, Luxol fast blue myelin staining and Bielschowsky silver impregnation.
- **Normal appearing white and grey matter from the stroke patients (NAWMS/NAGMS)**: Such areas were collected from regions, several centimeters distant from the focal ischemic brain lesions. Also, these areas of interest did not show neuropathological abnormalities using the same criteria as defined above.
- **Acute focal ischemic lesions (acute lesions) and their surrounding perilesional white and grey matter (Penumbra)**: Acute ischemic tissue damage was reflected by reduced numbers of neurons and glia cells, the remaining cells showing pale, sometimes fragmented nuclei and eosinophilic cytoplasm (Figure 1L). Such lesions, both in the grey and white matter, were characterized by reduced myelin staining in Luxol fast blue stained sections, loss of myelin associated glycoprotein (MAG) or cyclic nucleotide phosphodieserase (CNPase), associated with apoptosis like nuclear condensation in oligodendrocytes, but preservation of myelin oligodendrocyte glycoprotein or of major myelin proteins such as myelin basic protein or proteolipid protein within compact myelin sheaths (Figure 1A–H) 1. In general these lesions were pure ischemic lesions, although 4/9 of the grey matter but none of the white matter lesions contained variable amounts of perivascular erythrocytes, indicative of a hemorrhagic contribution (Figure 1D). Leukocyte infiltration in such lesions was sparse and mainly reflected by some increase in perivascular mononuclear cells. Besides of oligodendrocytes, a nearly complete loss of myeloid Iba‐1^+^ macrophages/microglia was also seen (Figure 1M). At the edge (penumbra), moderate edema was present but neurons, astrocytes, oligodendrocytes and myelin were preserved. However, there was an increased number of Iba‐1^+^ microglia‐like cells in the white and grey matter in comparison to NAWMC and NAWMS (Figure 1M,N).
- **Lesions in the resorption stage (early and late resorption stage)**: Such lesions were densely infiltrated by microglia and macrophages (Figure 1O), and were further classified by criteria, defined previously in multiple sclerosis lesions 6. Lesions in the early resorption stage were densely infiltrated by macrophages, which contained tissue degradation products reactive for Luxol fast blue (Figure 1K), or by immunocytochemistry for myelin proteins. Lesions at the late resorption stage showed a lower density of macrophages, which contained empty vacuoles or PAS positive degradation products (Figure 1L). Distinct separation between early and late resorption stage was observed in white matter lesions and in the deeper layers of the cortex, but was not seen in the outer cortical layers, due to the low content of myelin (Figure 1J).
- **Late cystic or scar lesion stage**: Such lesions with low macrophage or microglia content, presented as cystic cavities or astrocytic scar tissue.
- As a **positive control** for lymphocyte infiltration and their activation in inflammatory brain disease we also included a case of pathologically confirmed acute disseminated encephalomyelitis, who died 7 days after an acute measles infection within 24 h after onset of neurological disease 3. Thus, the time window between disease onset and death in this case was similar to that of patients with acute ischemic infarct lesions.

Overall, our material contained 15 lesions in the acute stage, 17 in the resorption stage and 4 in the late scar stage. The majority of patients with acute ischemic infarct lesions had died within 1 and 8 days after clinical disease onset. In those patients, which had acute ischemic infarct lesions despite a longer intervall between clinical onset and autopsy, a mixture of lesions with different lesion stages were seen (Table 1), suggesting progressive accumulation of lesions at different time points between disease onset and death.

### Immunohistochemical analysis

Immunohistochemistry was performed on paraffin sections with a biotin/avidin detection system as described in detail before 2, and outlined in Table 3. For control, sections were stained in the absence of primary antibodies and using isotype matched control antibodies.

**Table 3:** Antibodies and immunocytochemistry procedures.

#	Antibody	Origin	Target	Dilution	Antigen retrieval	Source
1	PLP	Mouse (mAB)	Proteolipid protein	1:1,000	St (E)	MCA839G; Serotec
2	MOG	Mouse (mAB)	Myelin oligodendrocyte glycoprotein	1:1,000	St (C)	42
3	CNPase	Mouse (mAB)	2′3′ cyclic nucleotide 3′ phosphodiesterase	1:2,000	St (E)	#SMI91; Sternberger Monoclonals
4	CD3	Rabbit (mAB)	T‐cells	1:1,000	St (E)	RM‐9107‐S; Neomarkers
5	CD8	Mouse (mAB)	MHC Class I restricted T‐cells	1: 250	St (E)	M7103; Dako
6	CD4	Mouse (mAB)	MHC Class II restricted T‐cells	1: 500+ 1:1000	St (E)	M7310; Dako DM119‐05;Acris
7	CD20	Mouse (mAB)	B‐cells	1: 100	St (E)	MS‐340; Neomarkers
8	PCNA	Mouse (mAB)	Proliferation Cell Nuclear antigen	1: 50000	St (C)	M0879; Dako
9	Iba‐1	Rabbit (pAB)	Ionized calcium binding adaptor molecule 1	1: 3,000	St (E)	019–19741; Wako
10	CD68	Mouse (mAB; IgG1)	CD68 110‐kD transmembrane glycoprotein in macrophages	1 : 100	St (E)	M0814; Dako,
11	HLA‐DR	Mouse (mAB; IgG1)	MHC Class II antigen	1:100	St (C)	M0775; Dako,
12	p22phox	Rabbit (pAB)	NADPH oxidase protein	1 : 100	St (C)	sc‐20781; Santa Cruz
13	TMEM119	Rabbit (pAB)	Transmembrane protein 119	1:100	–	HPA051870; Sigma‐Aldrich
14	P2RY12	Rabbit (pAB)	Purinergic receptor	1:2,500	St (E)	Harvard, Dr. Butovsky
15	HC10	Mouse (mAB; IgG2a)	Heavy chain of MHC Class I	1:2,000	St (E)	52
16	Ferritin	Rabbit (pAB)	Iron storage protein	1:1,000	St (E)	MO (F5012); Sigma‐Aldrich
17	CD206	Mouse (mAB; IgG1)	Mannose receptor	1:100	St (E)	ab117644; abcam
18	CD163	Mouse (mAB; IgG1)	Hemoglobin‐haptoglobin scavenger receptor	1:1,000	St (C)	NCL‐CD163; Novocastra
19	iNOS	Rabbit (pAB)	Inducible nitric oxide synthase I	1:200	St (E)	PA1–37925; Thermo Scientific
20	CD86	Goat (pAB)	Costimulatory T cell signal	1:250	St (C)	AF‐141‐NA; R&D Systems
21	CD31	Mouse (mAB)	Endothelial Cell,PECAM‐1	1:50	St (E)	M0823; Dako
22	vWF	Rabbit (pAB)	Von Willebrand Factor	1:500	St (E)	A0082; Dako

mAB = monoclonal antibody; pAB = polyclonal antibody; ST = antigen retrieval with steaming; E = EDTA buffer pH:8.5; C = citrate buffer; pH: 5.5.

**Figure 2:**
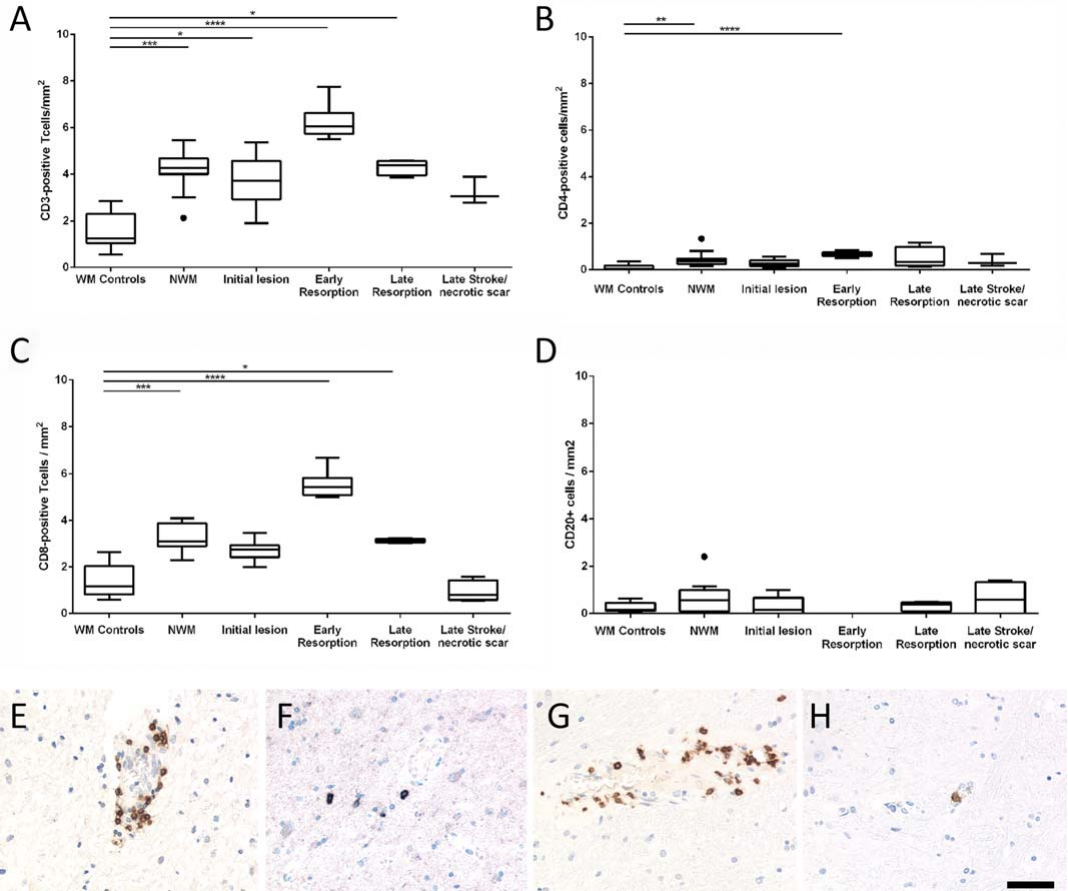
T‐ and B‐Lymphocyte infiltration in the normal white and grey matter of controls and in human ischemic infarct lesions. **A–D.** Only few T‐ and B‐lymphocytes were present in the brain tissue of age matched controls and in different stages of ischemic infarct lesions, defined in material and methods. However, the number of infitrating T‐cells (cells/mm^2^) was significantly increased, reaching its peak at the stage of early resorption (A–C), while this was not the case for B‐cells (D). The graphs show CD3^+^ cells (a), CD8^+^ cells (B), CD4^+^ cells (C) and CD20^+^ B‐cells (D). The lower panels show examples for lymphocyte infiltration in the lesions; the sections were stained for CD3: **E,** CD4; **F,** CD8: **G,** and CD20: **H.** Magnification bar: 50 μm.

**Figure 3:**
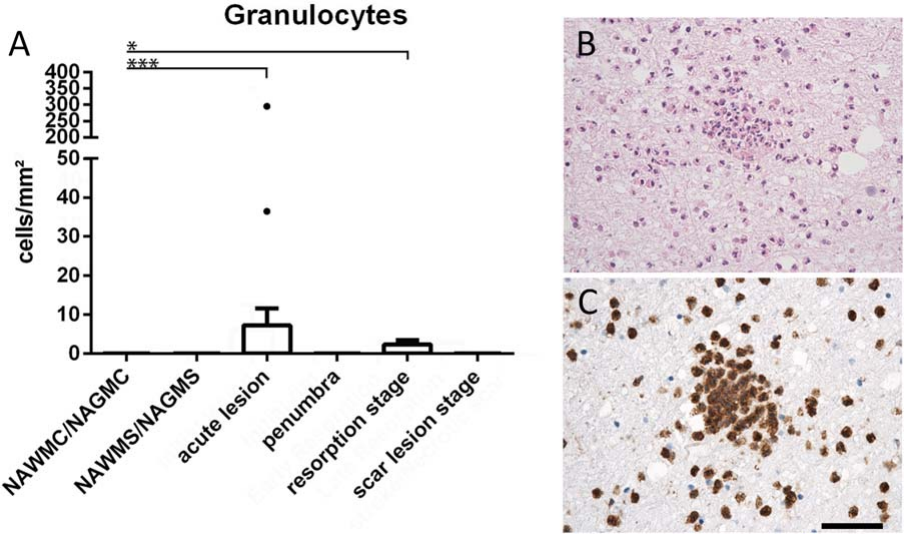
**A.** Tissue infiltration with granulocytes. In most of the cases, granulocytes were very sparse and this was the case during all lesion stages. Only a single case showed substantial numbers of granulocyte within the core of an acute lesion (single point with 295 cells/mm^2^). **B,C.** Show images of granulocyte infiltration in the case with substantial numbers of such cells, depicted in the staining with hematoxylin/eosin (B) and with p22phox (C). Magnification bar: 100 μm.

The primary antibodies used in this study were directed against different lymphocyte subsets and against monocytes, macrophages, microglia and granulocytes. The antibody source and dilutions as well as the necessary steps of antigen retrieval are outlined in Table 3. For the analysis of different inflammatory cells and their activation state the following markers and criteria were used:

- Lymphocytes: For evaluation of the basic phenotype of lymphocyte infiltration markers of all T‐cells (CD3), for MHC Class II restricted T‐cells (CD4), for MHC Class I restricted T‐cells (CD8) and for B‐cells (CD20) were included (Figure 2). When lymphocytes recognize their cognate antigen in the lesions, they proliferate and express the proliferating cell nuclear antigen (PCNA). After antigen specific stimulation T‐cells transiently express the nuclear transcription factor NFAT 32. PCNA and NFAT expression was analyzed by double staining with phenotypic T‐ and B‐cell markers.
- Granulocytes: Granulocytes were identified and quantified by their nuclear morphology (segmented cell nuclei). Since fragmented nuclei of apoptosis can sometimes mimick nuclear morphology of granulocytes 14, we confirmed our data by immunocytochemistry for p22phox (NADPH‐oxidase). This antigen is highly expressed in granulocytes (Figure 3), lower in activated macrophages or microglia and undetectable in other cells in human autopsy tissue. Thus, strongly p22phox^+^ cells with the size of granulocytes and segmented nuclei can easily be identified. We did not use other granulocyte markers, which in our experience are less reliable and sensitive in paraffin embedded human autopsy material.
- Differentiation between myeloid cells derived from recruited monocytes or microglia: This was performed by combining a pan microglia/macrophage marker (Iba‐1) with a new marker, selectively expressed on microglia derived myeloid cells [TMEM119; 4, 44]. TMEM119 is a transmembrane protein of unknown function, which is expressed on all resting (also called homeostatic) microglia in the rodent and human brain. During microglia activation, gene expression of TMEM119 is down‐regulated but protein expression on the cell surface remains preserved 44. This is the case irrespective of the morphological phenotype of the cells, such as resting microglia, ameboid microglia or macrophage like cells. In contrast, myeloid cells recruited into the lesions in the course of brain inflammation in rodents and humans do not express TMEM119 4, 44.
- States of microglia/macrophage activation: Recent studies defined the gene and protein expression profiles in different microglia stages. These results identified a profile for resting microglia, denominated as the homeostatic state of these cells in the normal brain 8, 11, 18, 25. From the panel of these different proteins we selected P2RY12, which is a purinergic receptor, sensing ADP release from injured cells. Its downregulation on mRNA and protein level is one of the most sensitive markers for the transition from resting to activated microglia 8. Activated microglia and macrophages can further differentiate depending to the type of injury and functional need 41. Specific markers for this functional transition, which we used in our study were CD68 (56), a marker for phagocytic activity, HLA‐DR, HC10 and CD86, markers for Class II and Class I restricted antigen presentation and T‐cell costimulation 23, 45, p22phox [NADPHoxidase, 15] and inducible nitric oxide synthase [iNOS; 51] as markers for oxidative activation, and ferritin as an activation marker related to iron loading of the cells 21. In addition, microglia and macrophages may differentiate into an anti‐inflammatory phenotype. To visualize this phenotype, we used the mannose receptor CD206 and the haptoglobin receptor CD163, which *in vitro* are induced in myeloid cells with anti‐inflammatory properties 36. All markers used in our study were extensively characterized regarding their reliability and expression patterns in human autopsy material of lymphatic tissue 62.
- In addition we used myelin markers (proteolipid protein, cyclic nucleotide phosphodiesterase and myelin olgodendrocyte glycoprotein) for lesion and lesion stage characterization and the vascular antigens von Willebrand factor and CD31 to determine the relation between inflammation to cerebral blood vessels.

Co‐expression of different macrophage and microglia markers was analyzed by double staining in the following combinations: Iba‐1, TMEM119, P2RY12, CD68, p22phox, CD163 and CD206. Proliferation of lymphocytes, macrophages and microglia was analyzed by double staining for T‐ and B‐cell markers or for Iba‐1, P2RY12 or TMEM119 with the proliferating cell nuclear antigen (PCNA). When primary antibodies were derived from different species, the same antigen retrieval techniques and incubation with primary antibodies was used as described above. Antibody binding was visualized with either alkaline phosphatase‐conjugated secondary antibodies or with biotinylated secondary antibodies and peroxidase‐conjugated streptavidin. Alkaline phosphatase or peroxidase reaction products were visualized by development with fast blue BB salt (blue) or amino ethyl carbazole (AEC; red), respectively.

Since both primary antibodies come from the same species (rabbit), double staining for P2RY12 or Iba‐1 with TMEM119 was performed with a different protocol 2 using extensive heat‐induced epitope retrieval between the subsequent immunohistochemical reactions. After deparaffination and a primary round of antigen retrieval (Table 3), sections were incubated with anti‐P2RY12 or Iba‐1, followed by biotinylated anti‐rabbit antibody and by avidin peroxidase and catalyzed signal amplification with biotinylated thyramine. Then, another round of antigen retrieval for 30 minutes at pH 9.5 was performed, which abolishes antibody reactivity from the previous round but leaves the amplified avidin binding intact, thus preserving the localized binding of Cy2 labeled streptavidin. Afterwards, the second immunohistochemistry reaction was applied using TMEM119 antibody as the primary antibody and (goat) anti rabbit Cy3 as a secondary antibody. For control, serial sections were stained with the identical protocol but omitting P2RY12, TMEM119 or Iba‐1 primary antibody. Immunofluorescence was visualized in a confocal laser microscope (Leica SP2).

### Quantitative evaluation

For quantification the following areas of interest were selected in the grey and white matter: Normal appearing tissue of controls, normal appearing tissue of stroke patients, acute ischemic lesion, the penumbra (edge) of acute lesions, lesions in the early resorption and late resorption stage and late cystic or scar lesions. The number of T‐cells and B‐cells were counted separately in the perivascular and parenchymal areas. Later, these values were pooled in the statistical analysis. Cells located within the vascular lumen, defined by the location of cerebral endothelial cells, were not counted.

For quantification of lymphocytes, a morphometric grid within the ocular lens was used and inflammatory cell numbers were manually counted in 10–20 fields of 0.25 mm^2^ per tissue area, spanning the entire lesion. In normal controls, randomly sampled areas of NAWM were quantified in the same way. All values are expressed as cell counts per square millimeter. Double stained cells with the proliferation marker PCNA and the activation marker NFAT2 with the T‐cell markers, CD3, CD4 and CD8, and the B‐cell marker, CD20, were quantified in the same way as described above.

For the quantitative evaluation of microglia/macrophages, sections stained were overlaid by a morphometric grid (0.2256 mm^2^) placed within the ocular lens and 2–3 fields per region of interest were selected. Cells, expressing the respective marker were counted and the values are expressed as cell counts per square millimeter.

Digital optical densitometry was performed for markers, which were not exclusively expressed in macrophages and microglia, such as the MHC Class I marker HC10 and ferritin, according to a previously published protocol 21. Expression of these markers was quantified by calculating the positive DAB signal area fraction using ImageJ. One to two images per ROI were taken at 10× objective lens magnification (0.43 mm^2^). Images were saved as TIFF. For digitally removing hematoxylin counterstaining, a color deconvolution plugin (freeware kindly provided from A.C.Ruifrok, NIH) was run. Further RGB images were converted into 8‐bit grey scale images and inverted. A threshold was set in resulting images and the area fraction was calculated. All values are expressed as percentage of positive area.

### Statistical analysis

Statistical analysis was performed with IBM SPSS and GraphPad Prism. Due to uneven distribution of our data, statistical analysis was performed with nonparametric tests. Descriptive analysis included median value and range. Differences between two groups were assessed with Wilcoxon‐Mann‐Whitney U test. In case of multiple testing (comparison of more than two groups), significant values were corrected with the Bonferroni‐Holm procedure. Interdependence of variables was evaluated by the Spearman non‐parametric correlation test. The reported *P*‐values are results of two‐sided tests. A *P*‐value ≤ 0.05 was considered statistically significant.

## Results

### T‐ and B‐cells are rare in human ischemic infarct lesions and do not proliferate

T‐ and B‐lymphocyte infiltration was analyzed in human ischemic infarct lesions in relation to that seen in acute disseminated encephalomyelitis (ADEM, inflammatory control with comparable short disease duration) and in normal controls. In ADEM, very high numbers of infiltrating T‐cells were present (168 CD3^+^ T‐cells and 146 CD8^+^ T‐cells per mm^2^). 19.8% of the tissue infiltrating CD8^+^ T‐cells expressed the proliferation marker PCNA and 14% the acute activation marker NFAT2. The number of lymphocytes in ischemic infarct lesions was extremely low in comparison to the inflammatory control, reaching maximal levels per mm^2^ on average of 6 cells for CD3^+^ T‐cells, 5.8 cells for CD8^+^ T‐cells, 0.5 cells for CD4^+^ T‐cells and 0.8 cells for CD20^+^ B‐cells. Despite their low numbers, a significant increase of CD3, CD4 and CD8 positive cells, but not of B‐cells was seen in ischemic infarct lesions in comparison to controls, which peaked in the early resorption stage (Figure 2). The dominant lymphocyte population within ischemic infarct lesions were CD3^+^ and CD8^+^ T‐cells. T‐ and B‐cells were located in the meninges and perivascular spaces, while diffuse infiltration of the lesioned tissue was occasionally seen with T‐cells. Proliferating T‐ and B‐cells and NFAT2 postive T‐cells were nearly absent with the rare exception of a single cell in single lesions or cases.

### Tissue infiltrating granulocytes are only seen in exceptional cases in ischemic infarct lesions

Granulocytes were present in variable numbers in the meninges and large perivascular spaces in 7/9 patients with acute infarct lesions and in 1/7 patients with later lesion stages only (Table 2). Within the ischemic infarct lesions, however, the number of tissue infiltrating granulocytes was very low, reaching densities of 0 to 11 cells/mm^2^ (Figure 3) and this was the case in patients, regardless whether or not the lesion contained a hemorhagic component or whether they were located in the white or grey matter. However, there was one case with an acute stroke, with 3 days of disease duration and a moderate hemorrhagic component, who showed a high density of PMNs in acute lesions (295 cells/mm^2^; Figure 3). No indication for an infectious cause of death was seen in this patient (Table 1).

### Characterisitics of microglia and macrophages in the normal appearing white and grey matter of controls and stroke patients

**Figure 4:**
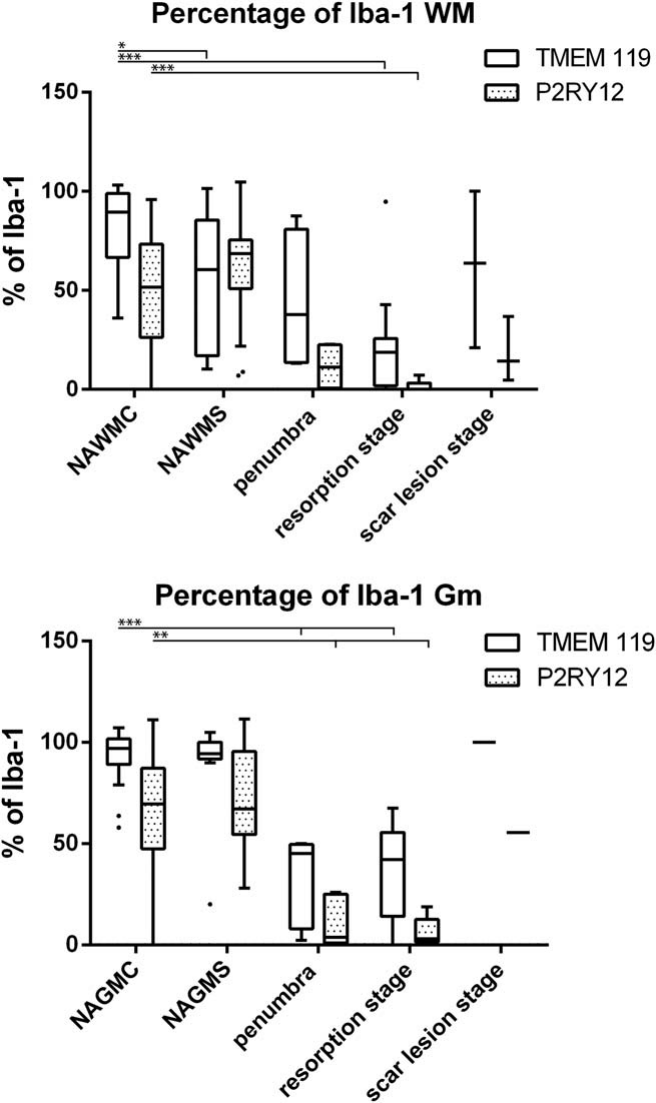
Percentage of Iba‐1^+^ cells expressing TMEM119 or P2RY12, in the brain tissue of controls and stroke patients and in different stages of ischemic infarct lesions. There is a significant reduction of the percentage of Iba‐1^+^ cells expressing these markers in the normal brain tissue of stroke and this percentage is further reduced within the lesions.

**Figure 5:**
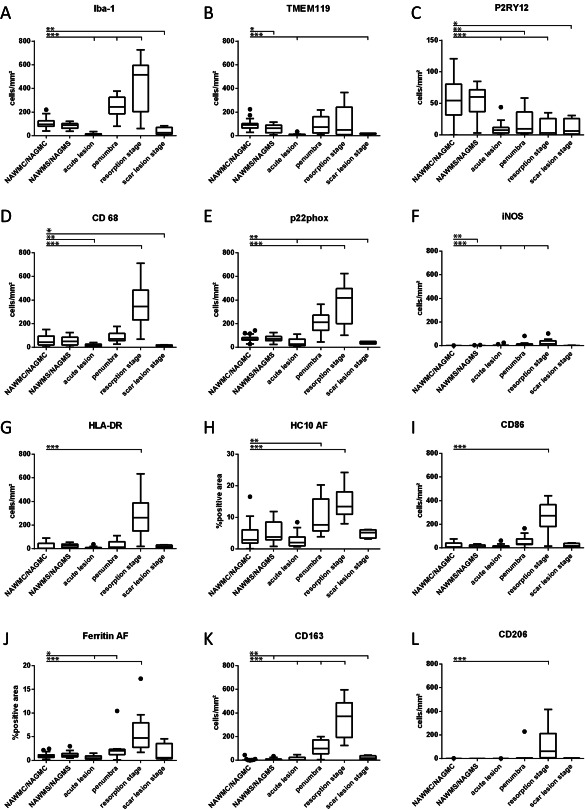
Quantitative profile of microglia/macrophage marker expression in the brain tissue of controls (WAWMC/NAWGS) and different stages of ischemic infarct lesions. For most markers, the values represent actual numbers of cells/mm^2^ in the different lesion areas. For the MHC Class I marker HC10 and for Ferritin (H,J), densitometry was performed, since these markers were also expressed on other resident cells of the brain. In these panels, the values represent the densitometric values, determined by area fraction (AF, see Material and Methods). *: *P* ≤ 0.05; **: *P* ≤ 0.01; ***: *P* ≤ 0.001.

White matter areas in control patients contained significantly more Iba‐1^+^ microglia or macrophages compared to grey matter areas (107.8 vs. 89.47; *P* = 0.014). TMEM119, a marker for resident microglia, was expressed on 97% of the Iba‐1^+^ cells in the grey matter and on 89% in the white matter (Figure 4). Overall, microglia in normal controls showed a partly activated phenotype, reflected by the expression of the homeostatic microglia marker P2RY12 in only 51% (WM) or 70% (GM) of Iba‐1^+^ cells and the expression of the NADPHoxidase complex protein p22phox on 75% of microglia in the white and grey matter (Figures 4 and 5). In addition, expression of the phagocytosis related antigen CD68 and the co‐stimultory molecule CD86 was seen, which was significantly higher in the white compared to the grey matter (*P* < 0.001). The expression of p22phox (NADPH oxidase) in microglia correlated significantly with the age of the control patients (*P* = 0.029), but we did not find gender‐dependent differences in microglia marker expression. Patients, who died under inflammatory or septic conditions did not show significant differences of microglia marker expression in comparison to those without inflammatory co‐morbidity

**Figure 6:**
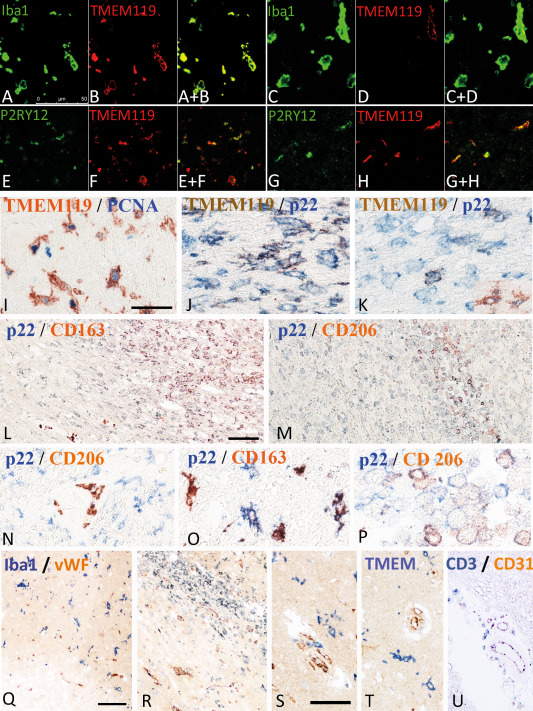
Double staining of microglia and macrophages with different phenotypic markers. **A–D.** Double staining with the pan microglia/macrophage marker Iba‐1 and the marker for resident microglia derived cells, TMEM119. While in the normal brain tissue in this case around acute stroke lesions the majority of cells co‐express both markers (A,B), the number of macrophage like cells expressing TMEM119 is low in lesions in the late resorption stage (C,D). The double stained images are shown in A+B and c+d. **E–H.** Double staining for the “homeostatic” marker P2RY12 with TMEM119 shows that a fraction of microglia derived cells in the penumbra of acute lesions (E,F) and in the late scar stage (G,H) co‐express both markers. The double stained images are shown in E+F and G+H. **I.** Double staining for TMEM 119 and the proliferation marker PCNA. Many microglia in the penumbra around acute ischemic infarct lesions show a nuclear expression of PCNA. **J,K.** Higher magnification of lesion areas presented in Figure 1O, showing partial double staining with TMEM119 and p22phox in microglia like cells at the ledge of the lesions (J), while the number of double stained cells in the center of the lesions is low (K). **L–P.** Double staining for the pro‐inflammatory marker p22phox and the anti‐inflammatory markers CD206 (M, N, P) and CD163 (L,O) in ischemic infarct lesions, in the early and late resorption stage. While pro‐inflammatory markers are mainly expressed in the perilesional areas and in the early lesion stages, the anti‐inflammatory markers are mainly present in the center of the lesions, depicting the late resorption stage; l: low magnification of the lesion edge; M = low magnification of the lesion center; N = perilesional tissue with p22phox expression in activated microglia and CD206 expression in perivascular macrophages; O = lesion edge with microglia cells, some of them being double stained; Q = lesion center with macrophage like cells, some only expressing p22phox, while others also express CD206. **Q–U.** Double staining for T‐cells, macrophages and microglia with the vascular markers von Willebrand factor and CD31: (Q) Acute ischemic infarct lesion with loss of microglia in the ischemic core (lower corner of the image) and acitvated microglia in the penumbra; there is no preferential accumulation of myeloid cells around the vessels; (R) Ischemic infarct lesion in the eraly resorption stage; there are few perivascular myeloid cells, but no preferential accumlation of these cells around smaller vessels. S–U. Perivascular versus parenchymal location of Iba‐1^+^ myeloid cells (s), TMEM119^+^ microglia (T) and CD3^+^ T‐cells (U); Iba‐1^+^ cells are seen in perivascular and parenchymal location, while TMEM 119^+^ cells are only present in the parenchyma; CD3^+^ cells are in the vast majority located around medium sized veins of the tissue. All magnification bars represent 50 μm.

A similar phenotype of microglia was also observed in the normal appearing white and grey matter of stroke patients (Figures 4, 5, 6).

### Macrophages in ischemic infarct lesions are derived from resident microglia and from recruited myeloid cells

In the necrotic core of acute lesions, a nearly complete loss of microglia was seen (Figure 1M). This was reflected in all microglia/macrophage‐specific markers used in the study (Figure 5). An increased density of Iba‐1^+^ cells was present in the penumbra zone of acute lesions in comparison to the NAWM (Figures 1M and 5A). On average 43% of the Iba‐1^+^ cells at the edge of acute lesions expressed TMEM119 suggesting their origin from the resident microglia pool (Figures 4 and 5B). In addition, a high percentage Iba‐1^+^ or TMEM119^+^ cells were double stained with the proliferation marker PCNA (median 45%; range 18%–57% at the lesion edge, decreasing toward a median of 4%, range 1%–6%, in the normal brain tissue at a distance of 2mm from the lesion edge) (Figures 1N and 6I). In lesions in the stage of resorption, the number of Iba‐1^+^, but not of TMEM119^+^ cells increased dramatically, suggesting substantial recruitment of myeloid cells from the circulation (Figures 4, 5A,B and 6C,D). This was more pronounced in the white matter (Median: 16% TMEM119^+^ of Iba1^+^ cells), while the proportion of TMEM119^+^ microglia derived cells was higher in the grey matter (Median 41% of Iba1^+^ cells). The total numbers of microglia and macrophages (Iba‐1) declined in the old scar lesions below the levels seen in the normal apearing tissue (Figure 5a). However, TMEM119^+^ cells remained throughout all lesions stages and their percentage in relation to total Iba‐1^+^ cells increased in old lesions in comparison to earlier lesion stages (Figure 4). These data suggest that in early stages of ischemic infarct lesions resident microglia are replaced in part by the proliferation of resident microglia in the penumbra, which migrate into the lesion, and in part by myeloid cells recruited from the circulation. Irrespective of their source, these cells phenotypically differentiate into macrophage like cells. In late scar lesions, recruited macrophages gradually disappear while some cells derived from the microglia pool persist and in part transform back into a homeostatic microglia phenotype. When grey and white matter lesions were analyzed separately, the above described patterns of microglia reaction and monocyte recruitment were similar (data not shown).

### Patterns of macrophage and microglia activation

Within the lesions, the total number of Iba‐1^+^ cells was increased (Figure 5A) but the percentage of Iba‐1^+^ cells expressing P2RY12 was significantly lower than in the normal appearing white and grey matter (Figure 4). However, most of the lesions, irrespective of their stage, contained a subpopulation of TMEM119^+^/P2RY12^+^ cells (Figure 6E–H). In addition, P2RY12 reactivity was not present on the entire surface of microglia, but showed punctate reactivity and the cells in part revealed a macrophage phenotype (Figure 6E,F). In the late scar stage of the lesions 50% of Iba1^+^ cells in the grey matter and 12% in the white matter expressed P2RY12 and in part morphologically resembled microglia, suggesting a partial conversion of these cells into a resting (“homeostic”) phenotype. Microglia and macrophages at the edge of acute lesions revealed a dominant pro‐inflammatory activation pattern (Figure 1O), with expression of molecules involved in antigen presentation (MHC‐Class I; Figure 5H) and the production of reactive oxygen or nitric oxide species (Figure 5E,F). This pro‐inflammatory activation peaked at the resorption stage and declined thereafter, reaching highest levels in the early resporption stage, defined by the presence of early myelin degradation products in macrophages (Figures 5D–J and 6J–M). On the contrary, the expression of molecules associated with an anti‐inflammatory phenotype peaked in lesions at the late resorption stage, where they were mainly present in macrophages in the center of the lesions (Figures 5K,L and 6L–P). Double staining revealed that many macrophage like cells, in particular at the late resorption stage, displayed an intermediate phenotype with co‐expression of pro‐ and anti‐inflammatory markers (Figure 6N–P). Overall, the lesion stage dependent activation patterns of microglia and macrophages were very similar in ischemic infarct lesions located in the grey or white matter of the brain (data not shown).

### Relation of the inflammatory reaction to blood vessels

Lymphocytic infiltrates were mainly present in the perivascular space of veins and venules (Figure 6U). T‐ and B‐cells were not accumulated around newly formed capillary sprouts within the penumbra or lesions. Double staining for myeloid markers with the endothelial markers von Willebrand factor or CD31 showed that the microglia reaction was homogeneous throughout the lesion without a preference in perivascular areas (Figure 6Q–T), however, TMEM119 negative Iba1^+^ cells in early lesions showed preferential accumulation around veins, but not around capillary sprouts (Figure 6Q–S). In contrast, TMEM119^+^ cells were only seen in the parenchyme, but not in perivascular location (Figure 6T).

## Discussion

Inflammatory mechanisms mediated by innate and adaptive immune responses have been suggested as important targets for stroke therapy. Many studies have shown the presence of inflammation in the lesions of experimental stroke models. Treatments, which interfere with microglia/macrophage activation or with brain infiltration by granulocytes or lymphocytes, presented beneficial effects in experimental studies. However, so far, the translation of these effects to human stroke patients has been disappointing 58. It, thus, appears necessary to provide a systematic account of the inflammatory response in human ischemic infarct lesions and to compare this with the data reported in experimental models. In our study, we analyzed the infiltration of lymphocytes, granulocytes and recruited monocytes and the activation state of microglia in different stages of the evolution of the human ischemic infact lesions. Our data show both similarities and differences compared to experimental models.

Many experimental studies have shown a prominent infiltration of granulocytes in infarct lesions 17, 27, 37, 47, 48. Taken together, they may appear already in the lesions after 30 minutes, reach their peak between 1 and 3 days and decline within the next days 29. Although prevention of their migration into the lesions reduced infarct size and mortality in experimental animals, at least in models of reperfusion 12, 31, 43, 61, a clinical trial in stroke patients was terminated due to lack of effect 58. In our study, we confirm the early infiltration of granulocytes into the meninges and, less prominently, into the perivascular space. However, the diffuse infiltration of the acute ischemic ínfarct lesion, as described to be profound in experimental models, was minimal, except for a single case, which showed massive infiltration in the lesions. This is in line with a study by Enzmann *et al* 14, where granulocyte extravasation was only seen in the meninges and not in the lesioned tissue. Discussing this study in an editorial 30, it was suggested that granulocyte extravasation in ischemic infarct lesions may depend on the severity of the ischemic tissue injury and the presence of a hemorrhagic component in the lesions. Perez de Puig *et al* supported this view by detecting extravasation of PMNs in fatal cases of human stroke lesions and in animal models and hypothesized PMNs access to the parenchyma from the perivascular space and the leptomeninges after prolonged occlusion through activation leading to NETosis 40. However, in our study, complete tissue necrosis has been seen in the center of all cases with acute lesions, and diffuse granulocyte infiltration of the lesions was also largely absent in most cases with a hemorrhagic component. The presence of high numbers of granulocytes in one case can so far not be explained on the basis of lesional timing or stage, nor of specific pathological lesion phenotype, nor by the clinical course, such as for instance infectious co‐morbidity.

Lymphocytes have been identified in experimental stroke lesions in low numbers, being mainly present in the later stages of ischemic injury 9, 28, 29, 53. Flow cytometric analysis showed a dominance of the CD4^+^ subset 19, 35. B‐lymphocytes have rarely been analyzed, and their total numbers were similar to those of T‐cells 35, 53. Several possible roles of B‐cells have been postulated in stroke patients, having disease enhancing as well as protective effects 49. Treatments targeting lymphocytes have been tested in stroke patients, describing some positive effects for fingolimod, a drug which may also have direct neuroprotective actions 34. So far, no convincing benefit was seen with natalizumab, which blocks the passage of leukocytes through cerebral vessels 13, 58. In our study, the numbers of T‐ and B‐cells within the lesions were very low, although they showed a significant increase for T‐cells in comparison to controls. Similar to those present in human multiple sclerosis or control brains 5, 22, the recruited T‐cells were mainly CD8^+^. Their numbers peaked at later stages of brain ischemia, suggesting recruitment in parallel with other leukocytes, such as macrophages. In the absence of proliferation and NFAT expression, it is unlikely that the cells are activated by their specific cognate antigen in the central nervous system. Thus, these data suggest that T‐ and B‐cells are secondarily recruited into the lesions, and that their local contribution to the pathophysiology of tissue damage is minor or absent. This does, however, not exclude that systemic adaptive immune responses in the course of stroke evolution may modify the disease 10.

Experimental models suggest that microglia and recruited macrophages play an important role in the development of infarct lesions. Similar as described in experimental models 60, we found that microglia are lost from the ischemic core of the lesions, but that there is a profound microglia activation and proliferation in the surrounding (penumbra) area. Using TMEM119 as a specific marker for resident microglia 4, 44, we found that a substantial proportion of macrophage like cells in the lesions during the early resorption stage is derived from the original microglia pool. A similar conclusion has been reached in experimental models using radiation bone marrow chimeras 46, 54. With further lesion maturation, the percentage of TMEM119^+^ cells decreased, either suggesting a progressive loss of microglia derived macrophages in advanced lesions or a partial downregulation of TMEM119 in microglia after activation and transformation into macrophages 8, 44.

A major difference in microglia phenotype between humans and rodents is seen in the normal brain and in relation to brain aging. In rodents, a set of homeostatic markers including P2RY12 is expressed in microglia in the normal brain and activation antigens involved in phagocytosis, antigen presentation or oxidative injury are rare or absent. 7, 8. In humans, this homeostatic phenotype in microglia is partially lost in the normal white and grey matter of aging controls 62. These differences between humans and rodents have been recently confirmed in a large gene expression analysis, showing fundamental differences in microglia activation in the normal brain and during aging between humans and mice 16.

In human ischemic infarct lesions, microglia activation is further enhanced, possibly related to the neurodegenerative and/or inflammatory environment 7, 8. However, there was still a substantial proportion of microglia expressing P2RY12 in the penumbra of acute infarct lesions and in the early resorption stage. P2RY12 is a purinergic receptor, which senses ADP release 24 and which is involved in the uptake of cell processes in the course of synapse remodeling 50, and also in ischemic lesions. Blockade of P2RY12, by genetic deletion in microglia or by pharmacological antagonists reduces neuronal injury in experimental stroke lesions 59. In addition, the vast majority of macrophages in the lesions, irrespective of their microglia or monocyte origin, expressed markers for pro‐inflammatory functions, such as antigen presentation, phagocytosis and oxidative injury. Furthermore, molecules involved in controlling iron homeostasis were highly up‐regulated, which may in part reflect the age related iron accumulation in the normal human brain and its liberation in lesions of a diseased brain 21. On the contrary, antigens which are associated with anti‐inflammatory activation of macrophages, such as CD163 and CD206, peaked at later lesion stages (late resorption stage), which is in part different in timing as described before in experimental models 26, 55. In peripheral blood, the ratio between pro‐ and anti‐inflammatory macrophages may predict clinical course and prognosis in human stroke patients 57.

Besides a spectrum of different causes of the infarct lesions and different co‐morbidities the patterns of lymphocyte, granulocyte and monocyte infiltration into the lesions and of microglia activation was very similar, when a staging of the lesions was performed on neuropathological criteria. Since all patients had a very severe disease and died in the course of the disease, it was not possible to determine, whether the degree of inflammation in the lesions influenced disease severity. Furthermore, the patterns of microglia and macrophage activation was very similar between large and small ischemic infarct lesions. Our data suggest that in the initial phases of acute ischemic infarct lesions microglia are destroyed in parallel with neurons and other glia cells. The lesion is then infiltrated by macrophages, which are in part derived from resident microglia cells, which proliferate in the penumbra and migrate into the lesions. In addition, however, there is a substantial recruitment of monocytes from the circulation that peaks during the resorption phase of the lesion. Irrespective of their origin, the cells phenotypically differentiate into macrophages, which are essential in the removal of tissue debris. Such macrophages and the microglia in the surrounding tissue show primarily a pro‐inflammatory phenotype, but transform with lesion maturation into an intermediate pro‐ and anti‐inflamatory phenotype that temporally may be associated with reparative phenomena in the lesions, such as remyelination. Finally, hematogeneous macrophages disappear from the lesions, while cells with a phenotype of resting homeostatic microglia reappear within the old gliotic scar tissue. In contrast, the contribution of granulocytes, T‐cells and B‐cells was much less pronounced than expected. Species related differences in the inflammatory response may in part account for this. There is, however, an additional difference between human stroke and its experimental models. To induce stroke lesions in rodents, quite extensive surgical intervention is required in comparison to the spontaneous induction of the lesions in humans. Surgical trauma can cause systemic activation of the innate immune system, which also may lead to non‐specific activation of adaptive immune responses 39. This should be kept in mind as a caveat, when translating effects of immunomodulatory treatments in experimental models to human therapy. Thus, anti‐inflammatory therapy in human stroke patients may be beneficial, when it targets specifically pro‐inflammatory activation of macrophages and microglia in the early stages of the lesions, but leaves their function in debris removal or neuroprotection intact.

## Conflict of Interest

The authors have no conflict of interest.

## Authors Contributions

TZ, JMS, SC: generation of all quantitative data; CB: generation of clinical data; HLW and OB: expertise on microglia biology, design of the study; critical evaluation and discussion of the results; HL: design of the study, neuropathological case selection and analysis, supervision of all immunocytochemical and quantitative data generation; All authors have extensively contributed to the preparation of the manuscript and the interpretation of the findings.
